# Detecting disease associated biomarkers by luminescence modulating phages

**DOI:** 10.1038/s41598-022-06433-y

**Published:** 2022-02-14

**Authors:** Janne Kulpakko, Vilhelmiina Juusti, Antti Rannikko, Pekka E. Hänninen

**Affiliations:** 1 Aqsens Health Ltd., Itäinen Pitkäkatu 4B, 20520 Turku, Finland; 2 Laboratory of Biophysics and Medicity Research Laboratories, Institute of Biomedicine, Faculty of Medicine, University of Turku, Tykistökatu 6A, 20520 Turku, Finland; 3 Department of Urology, Helsinki University, Helsinki University Hospital, Helsinki, Finland

**Keywords:** Biochemistry, Biophysics, Molecular medicine

## Abstract

Assessment of risk for a given disease and the diagnosis of diseases is often based on assays detecting biomarkers. Antibody-based biomarker-assays for diseases such as prostate cancer are often ambiguous and biomarker proteins are frequently also elevated for reasons that are unspecific. We have opted to use luminescence modulating phages for the analysis of known acute inflammatory response biomarker CRP (C-reactive protein) and biomarkers of prostate cancer in urine samples. Firstly, CRP was used to simulate the detection process in a controlled chemical environment. Secondly, we tried to classify more challenging lethal prostate cancer samples from control samples. Our unique method utilizes a special biopanning process in order to create special phages capable of capturing a dye necessary for detection and potential biomarkers. As the biomarker-molecules interfere with the phages, dye is repelled from the phage network resulting in an altered reporter luminescence. These changes can be observed with an absorbance reader and even with the naked eye. The simple method could present an alternative for screening of disease biomarkers. For prostate cancer urine samples, we achieved a sensitivity of 80% and specificity of 75% to detect Grade Group (GG) 4 and 5 prostate cancer.

## Introduction

Preventive healthcare optimally relies in addition to healthy lifestyle promotion and education of the population also on early diagnostics and screening of common diseases with significant public health impact. For example, screening coupled with proper treatment of communicable diseases like tuberculosis have resulted in outcomes that have changed the disease landscape permanently. Yet diagnosis of non-communicable diseases often occurs only after the patient has experienced symptoms. Also, diagnostic methods are not always compatible with mass-screening purposes yielding a delayed treatment window starting later than desired for optimal outcomes from the patient and healthcare system point of view.

Prostate cancer (PCa) is the most common cancer in men. Prostate specific antigen (PSA) based screening and early detection of PCa is challenging because it leads to significant overdiagnosis and potentially also overtreatment of clinically insignificant low-risk disease. This increases the economic burden on the healthcare system while it does not improve survival of the patients but may reduce the quality of life of the patients. Therefore, there is an unmet need for new biomarkers to detect clinically significant PCa^1,2^. Currently, PSA is the most commonly used diagnostic biomarker for PCa screening and early detection—with known deficiencies. Magnetic resonance imaging (MRI) is increasingly used for prostate cancer risk stratification before biopsy. However, it is a relatively laborious and costly process and subject to significant inter-reader variability thus hampering it’s use as a first line PCa screening test. Therefore, novel specific biomarkers for clinically significant prostate cancer measured with a simple assay would be desirable for population based PCa mass screening campaigns.

Typical biomarker assays rely on the use of luminescence—the luminescence signal is measured either as a result of a chemical reaction or as a photo-induced signal. Our choice, Lanthanide based photo-luminescent reporters are optimal in many ways because they provide an extremely sensitive, practically background free measurement option in gated, time-resolved signal acquisition mode. Furthermore, the Lanthanide ion itself has a very low absorption coefficient and requires a sensitizing “antenna”-moiety to be detected. This “antenna”-concept allows us to modify the luminescence by modulating the chemical environment that creates or cancels the antenna-effect in the unknown sample^3^. The unique properties of lanthanide complexes as sensors and reporters are also widely studied providing ample literature from sensitizer-lanthanide configurations and ideas for various assay versions with different sensitivities and stabilities in different applications^4^.

An emerging and rapidly growing, but historical alternative for biosensing applications is the use of phages (also known as bacteriophages). They are low-cost and easy to manipulate and present an alternative platform for biomarker detection^5,6^.

In our earlier work we have shown that the use of sensitive lanthanide reporters together with phages has opened a new track for development of scalable screening tests for clinical samples^7^. Our biosensing system utilizes the inherent environmental sensitivity of lanthanide luminescence that is “tuned” to differentiate samples by interacting with luminescence modulating components.

In the present work, we describe a biosensing method based on reporter luminescence modulation utilizing quencher dye molecules—with the surprising observation that the reaction may also be followed with the naked eye, when analyte concentration is high. The system is based on dual-affinity biopanned phages capable of binding to the freely moving quencher dye, and at the same time binding to our target biomarkers—the reporter molecule is then non-specifically acting with the phage-dye-analyte-complexes creating an observable signal change as a function of the analyte concentration. The biopanning towards the disease specific biomarkers takes place with a representative pool of patient samples. Our approach provides flexibility beyond known methods firstly by allowing detection of any disease specific biomolecules within the affinity limits of the phages and secondly, through the short development phase, with PCa involving biopanning with actual Grade Grouped patient samples. Whereas our reporter-lanthanides have outstanding properties for detection, the second observed property from our studies, absorbance difference, offers a possibility to detect diseased samples in a simple, potentially mobile platform as the reaction can be measured e.g. with a simple absorbance reader or camera that analyses the color intensity of the image taken from the well—in some cases, with proper control color comparisons a simple yes/no answer can also be given directly by the observer.

In this work, we first demonstrate how an assay for C-reactive protein (CRP) can be created, simulating a generic biomarker in our system. Secondly, we apply our system to a more complex challenge, i.e., diagnose PCa in urine samples. Our ultimate aim was to separate samples from patients with potentially lethal GG 4–5 PCa from clinically insignificant and benign samples.

## Methods

### Reagents and materials

CRP (pc: AG723), Europium(III) chloride hexahydrate (CAS: 13759-92-7, pc: 212881), Glycine (CAS: 56-40-6, pc: G7126), Resazurin sodium salt (CAS: 62758-13-8, pc: R7017), Sigma 7-9^®^ (Tris) (CAS: 77-86-1, pc: T1378), Sodium chloride (CAS: 7647-14-5, pc: 746398), 4,4,4-Trifluoro-1-(2-naphthyl)-1,3-butanedione (NTA, CAS: 893-33-4, pc: 343633), Trioctylphosphine oxide (TOPO, CAS: 78-50-2, pc: 223301), Tryptic soy broth (CAS: na, pc: 22092) and Tween-20 (CAS: 9005-64-5, pc: P1379) were purchased from Sigma-Aldrich (St. Louis, MO, USA). Dimethyl sulfoxide (DMSO, CAS: 67-68-5, pc: D/4121/PB15) and HCl (CAS: 67-68-5, pc: H/1150/PB17) were purchased from Fisher Scientific (Hampton, USA). Bovine serum albumin (BSA, CAS: 9048-46-8, pc: P6154) was purchased from Biowest LLC (Riverside, MO, USA). Clear C-Shaped Immuno Nonsterile 96-Well Plates were purchased from ThermoFisher Scientific, Waltham, MA, USA. Brilliant green (CAS: 0633-03-04, pc: 229601000) from Acros Organics was used as a dye and Ph.D.-12 phage display library (pc: E8110S) from New England Biolabs as a phage library for two-step biopanning process. *E. coli* strain K-12 was used for phage amplification. Lignin chips were used to immobilize the dye molecules but also a lignin-like polymer, such as an amorphous, irregular three dimensional and highly branched phenolic polymer, can be used for the same purposes. Chips used in the first biopanning round were made of a mixture of highly branched phenolic polymers and cellulose.

#### Clinical samples

Ethical approval for the use of urine samples and clinicopathological data was obtained from the Institutional Ethics Committee of the Hospital District of Helsinki and Uusimaa (HUS/3372/2019 for the PASSIONATE study and HUS/850/2017 for the DEDUCER trial). PASSIONATE study is a retrospective registry-based study utilizing data and samples S obtained from patients participating in the Helsinki Biobank. DEDUCER trial is an ongoing prospective clinical trial consenting patients with urological malignancies in the HUS Helsinki University Hospital. PCa diagnostics was based on prostate MRI as a triage test followed by targeted ± systematic prostate biopsies. All experiments were performed in accordance with relevant guidelines and regulations. Informed consent is obtained from all the participants and research is performed in accordance with the Declaration of Helsinki.

### Development of the novel diagnostic test

**Figure 1:**
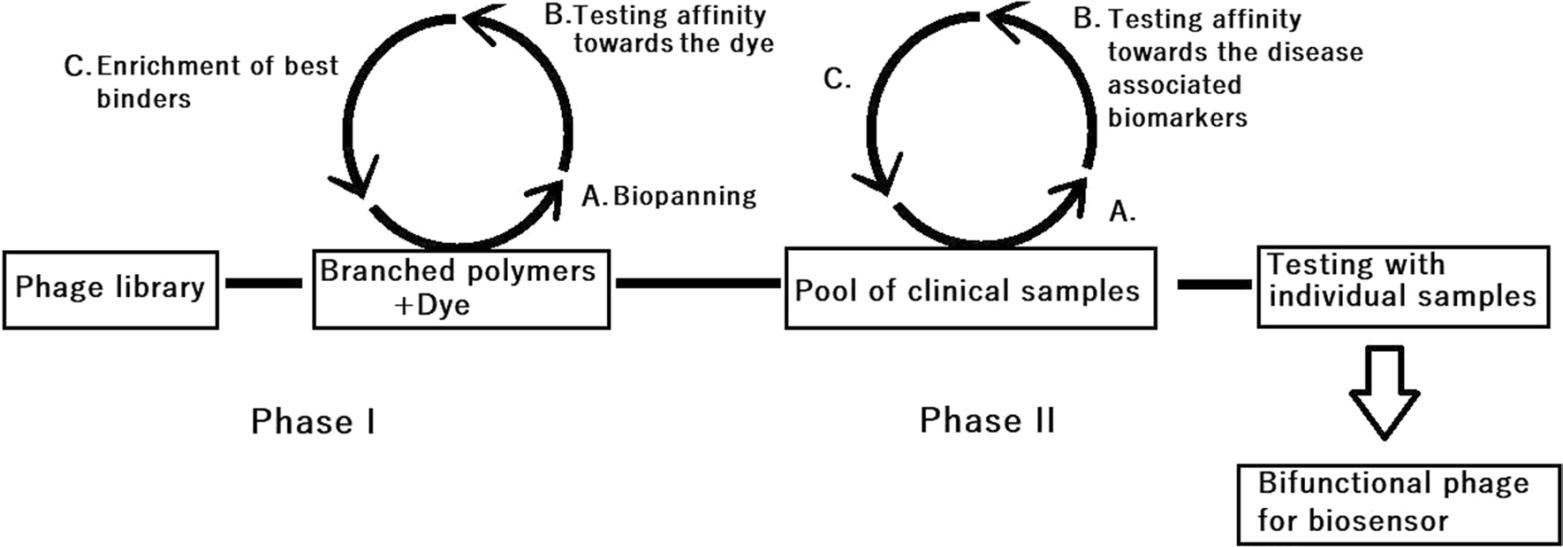
Schematic diagram of two-phase biopanning process and creating interaction between the phage, dye and the biomarkers of interest, such as ones in lethal PCa urine samples.

First step of developing a biosensing system consists of selecting dye/quencher binders from the phage library. The dye was immobilized to chips made of a mixture of highly branched phenolic polymers and cellulose. Binding phages were selected from this surface (Fig. 1). Binders from the first biopanning stage were used in the second biopanning stage against a simulant biomarker CRP and later, to test the suitability for intended application, a pool of PCa patient samples. Phage clones were tested with sensitive lanthanide reporters in the presence of dye and CRP or patient samples. From a tested group of clones the best interacting phage was enriched for both applications. The function of the chosen phage was further tested with a CRP dilution series and individual patient samples. Finally the accomplished data was compared to the concentrations of the biomarker and the clinical diagnosis of the patients.

#### Biopanning stage 1

Before the biopanning experiments the chips were rinsed with distilled water and the dye was immobilized to the surface with a saturated solution. Then the chips were washed, blocked with 2% BSA and left overnight at + 4 °C. After washing, they were autoclaved 120 °C for 60 min and stored at RT. The biopanning system, The Ph.D.-12 phage display peptide library containing 1.5 × 10^13^ plaque-forming units (pfu)/ml had required complexity of 2 × 10^9^. A 10 μl aliquot of the random peptide library was incubated with chips covered with brilliant green at RT for 30 min with gentle shaking in a microcentrifuge tube containing 1 ml of physiological saline. Unbound phages from brilliant green chips were washed serially with 4 ml of TBSTbuffer [0.1% (v/v) Tween-20 in TBS (50 mM Tris–HCl, pH7.5, 150 mM NaCl)]. After the 4 to 8 washes, the bound phage was eluted with 1 ml of 0.2 M glycine–HCl (pH 2.2). In each round, the bound phages were rescued and amplified to make more copies. Altogether, 3 rounds of the first biopanning stage were performed to achieve adequate affinity towards the dye. Affinity was tested parallelly by comparing the formation of phage-dye complexes with the random phage library and the ones from biopanning step one. These brilliant green binding phages were used in a second round of biopanning against both CRP and pooled disease samples.

#### Biopanning stage 2

The second set of biopannings were done first for CRP and then for lethal PCa samples. Both biopannings were performed following the same protocol described below. Before biopannings, CRP was diluted to 200 mg/L in physiological saline and 1 to 10 diluted lethal PCa urine samples were pooled (n = 5). These dilutions were added to microplate wells for 6 h in + 4 °C. After washing, the wells were blocked with 2% BSA and left overnight at + 4 °C. Ready wells were used for three (CRP) and four (PCa) rounds of biopanning (three rounds of 10 washes) and the bound phages were harvested and multiplied for luminescence assay testing the functionality in the assay system.

#### Testing procedure

CRP was diluted to physiological saline as clinically relevant concentrations to form a dilution series. The urine samples were centrifuged at 10,000 rpm for 5 min and the remaining clear supernatant was then diluted 1 to 50 in physiological saline. For measurement, each dilution or sample was divided as three replicates in 100 μl volume per well to a 96 well plate. Before adding the samples, brilliant green (6 mM) and phage solution (4.0 × 10^9^ pfu) or chemical modulator, resazurin sodium salt (185 µM), were added to the wells. After adding the sample, the reporter solution was added to the wells. The concentrations of the reporter components were 3.6 µM of Europium chloride hexahydrate, 2.1 µM of NTA and 2.1 µM of TOPO in assay with brilliant green and phage solution and respectively 3.7 µM, 2.2 µM and 2.2 µM with the chemical modulator. After 10 min of incubation, luminescence emission intensities were measured in a 400 μs window after a 400 μs delay time using Spark multimode microplate reader (Tecan, Switzerland). After 120 min incubation, absorbance was measured at 623 nm using same reader.

## Results and discussion

### The biosensing system

**Figure 2:**
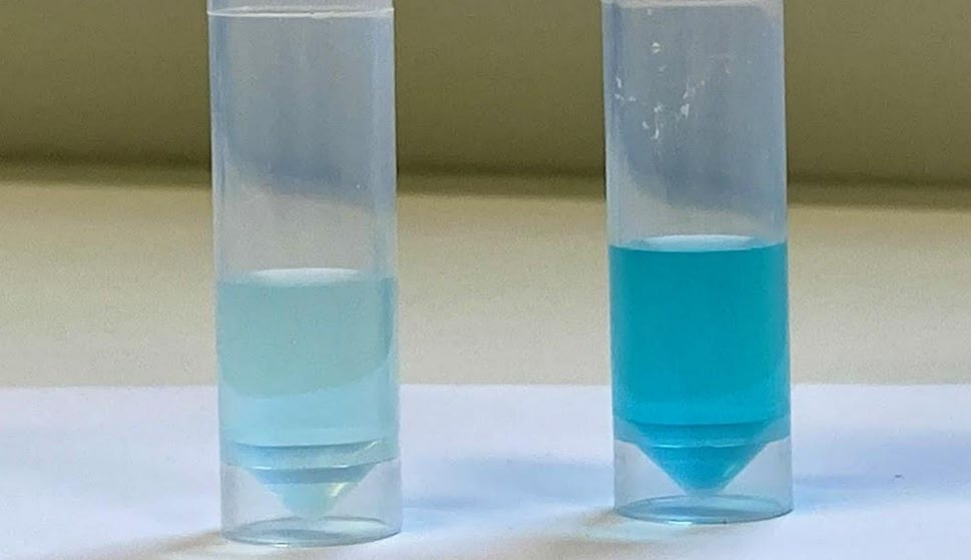
Illustration of quencher binding properties of biopanned phages. Right tube shows a mixture of quencher (brilliant green) and phages (not specific for brilliant green) in a physiological saline solution. The left tube shows the same concentrations of quencher and three cycle biopanned phages against the quencher molecule.

Phage-based constructs biosensing is a rapidly emerging biosensor technology covering a wide range of applications. In this study we created a biosensing system that started in the first phase by modifying the phage to bind the chosen quencher molecule (Fig. 1). The phage was biopanned against a quencher that was immobilized to a polymer mixture (A). After several rounds of biopanning procedures, a phage clone against the quencher was attained (B–C) 2. As a result of binding to these phages, the quencher loses some of its absorbance properties. The absorption difference is clear between specifically dye-binding and non-binding phages in equivalent solutions (Fig. 2). The loss of absorption and thus the quenching capacity can also be observed using a lanthanide reporter and time-resolved measurement, as is demonstrated in our CRP and PCa assays, with significantly better sensitivity than that of absorbance.

At the second stage of creating a biosensing system, the molecules of the target-sensing solution were bound to the bottom of the microplate wells. In the first study, the coating solution was CRP in physiological saline. In the second study, the coating solution was a pool of clinical GG 4–5 PCa urine samples, presumably containing disease specific, yet unknown biomarkers. Our hypothesis was that urine would contain PCa biomarkers and some of these biomarkers would be present in abundance in the samples for potentially lethal PCa compared to non-lethal and benign ones. Potential biomarkers derived from prostate cancer cells are gradually released into prostatic fluids and then into the urinary tract. As an example, several research groups are studying non-PSA urinary biomarkers of clinically-significant prostate cancers like HOXC6 (Homeobox C6), DLX1 (Distal-Less Homeobox 1), PCA3 (Prostate cancer antigen 3), ERG (ETS-related gene), SPDEF (SAM pointed domain-containing Ets transcription factor), and TMPRSS2 (Transmembrane Serine Protease 2)^8–10^.

The enriched phages from the first phase of biopanning were biopanned towards the biomarkers bound to the surface of the microplate wells at the second stage. After several rounds of biopanning, a clone detecting GG4-5 samples was enriched. At this second phase, it is notable that depending on the biomarker, it may either (a) competitively substitute the dye bound on the phage or (b) bind to the phage together with the quencher dye. In either case, a clear change in arrangement of dye-reporter configuration (and absorption) can be observed.This phage clone was used in the final biosensing system.

The binding of phages with the quencher molecules results in a clear decrease of absorption as also observed with the naked eye. The change is likely due to the change in the microenvironment of the dye^11^. Binding of the target biomarker further enhances or reduces the effect depending on the biopanning result. Therefore, it is the critical point of the system, but engineerable with several techniques.

**Figure 3:**
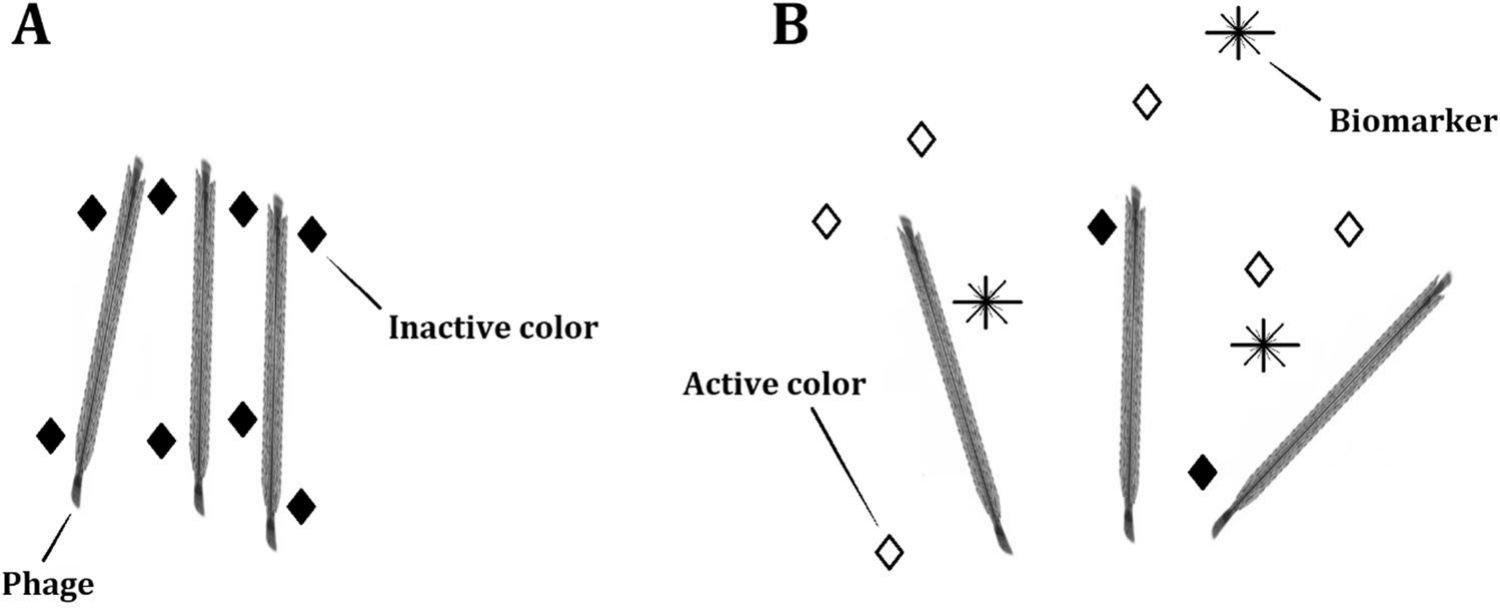
Possible reaction mechanisms for dye and biomarker binding to the network of specific phages. In the left side of the figure, the biomarker is not present and the phages are organized along dye molecules. The right side of the figure shows the displacement of dyes from the vicinity of the phages (**A**). Another possibility is the direct interaction of dye-phage complexes to the biomarkers (**B**).

For the reaction there are two possible explanations (Fig. 3). First, the phage binds dye and the color is lost (dark rhombuses) seen in the left side of the figure. As the biomarker (stars) reaches the phage binding area, it displaces dye molecules nearby by changing the microenvironment and phage network (a). This results in free dye molecules having normal color characteristics (light rhombuses). Second possible explanation is that dye stays in the phage when the biomarker binds to the vicinity of dye molecules (b). This interaction may change spectral characteristics as Karukstis et al. observed.

### Detection of CRP

In order to demonstrate our system in a controlled setting we used CRP as a model biomarker. The outcome of the two-stage biopanning in this case was a biosensing system capable of recognizing both the brilliant green and CRP. The results were comprehensible and supported our hypothesis of how the possible mechanism of reaction happens between all the components present in the well. Notably, due to the pentameric structure of the CRP, phages may also be ”bridged” together—potentially enhancing the effect if displacing the dye from the phage^12^.

**Figure 4:**
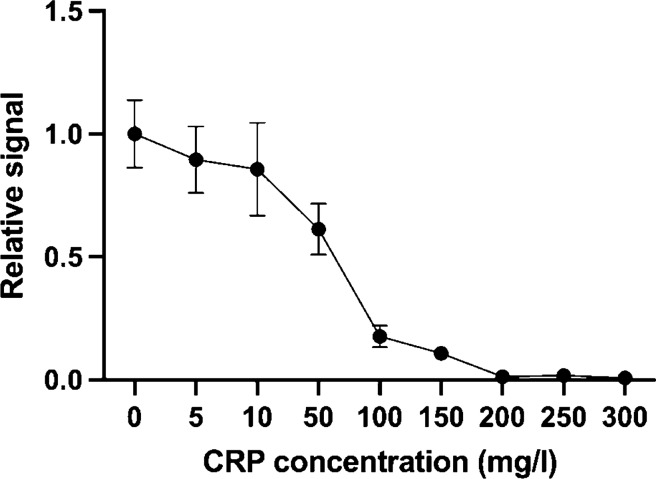
The CRP measurements with reporter luminescence. The relative luminescence is decreasing as a function of CRP concentration. Error bars are the coefficients of variance % of the signals from three replicate measurements.

As shown in Fig. 4, the CRP biomarker in the sample resulted in a decline of relative reporter luminescence. The strongest decline with our set-up is seen around 20 mg/L of CRP which is within the clinically relevant area. An average level of CRP in serum in a healthy person is around 0.8 mg/L but increases as much as to 1000-fold in different types of infections and inflammations^13^. The assay performance within clinical levels demonstrates the convenience of the system considering the development of the test took only a few days. However, it is important to remember that the demonstration test was performed in pure physiological saline lacking any interfering components inherent to clinical samples used for biomarker measurements.

**Figure 5:**
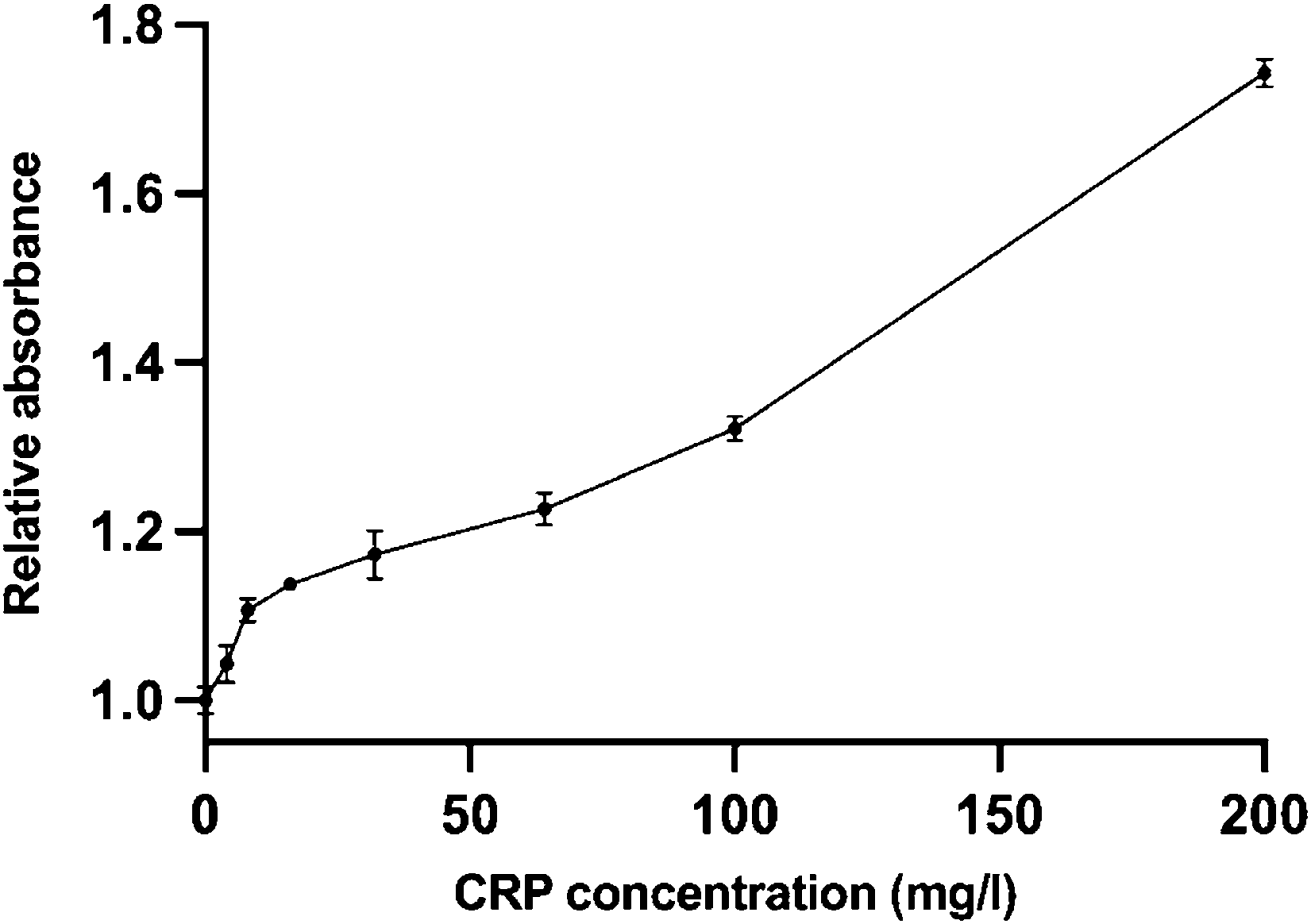
The CRP measurements with absorbance after 1 h of incubation in physiological saline. The relative absorbance is increasing as a function of CRP concentration. Error bars are the coefficient of variance % of absorbance from three replicate measurements.

In Fig. 5, CRP presence in a sample resulted in an increase of relative absorbance. The strongest increase is seen from 0 to 100 mg/L of CRP. The effect is opposite when compared to the measurement of reporter luminescence. The luminescence decreases when phage interacts with the CRP and dye is repelled to a solution and is able to quench the luminescent reporter. On the other hand, the absorbance of the solution increases, when phage binds to the CRP and dye is repelled.

### Detection of Grade-Group 4–5 prostate cancer

The true test for our biosensing system was to separate potentially lethal, GG 4–5 PCa cases from clinically insignificant GG 0–3 PCa. This means that after achieving brilliant green binding capabilities the phage has to reach a reasonable affinity towards biomarkers or factors that urine from patients with potentially lethal PCa contains.

In general, biomarkers are not perfect. For instance, it is known that advanced prostate cancers can eventually lose PSA expression and therefore PSA is no longer a usable biomarker^14^. PSA also has several different molecular forms which, when analyzed, may provide more precise information. Also, new candidate biomarkers for PCa, such as mRNA and DNA methylation based biomarkers have been suggested. Some of the biomarkers have been suggested to be preferably associated with aggressive clinically significant PCa^18^. Compared to blood samples urine samples are less complex and require less pretreatment before biomarker analysis. For instance, urine metabolite analysis performed with Liquid Chromatography–Mass Spectrometry (LC–MS) and Nuclear Magnetic Resonance (NMR) spectroscopy have revealed certain metabolic patterns related to breast and ovarian cancers compared to healthy controls. It was observed that the concentration of most metabolites in urine was lower in patients with breast cancer than in healthy controls^15,16^.

**Figure 6:**
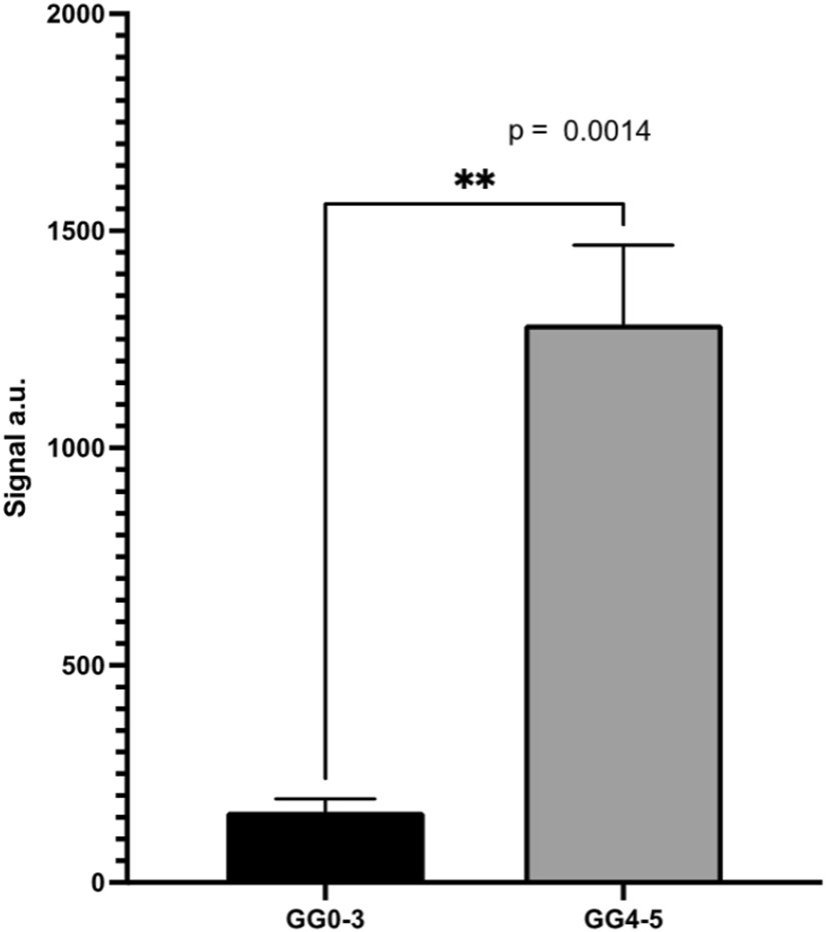
The phage-based assay signal for the potentially lethal and non-lethal PCa. The Y-axis is the linear combination of the measurements (Mean, N = 96, median of 3 replicates, error bars SEM). The left bar shows PCa GG 0–3 (N = 90) samples and the right bar shows GG 4–5 (N = 6). Grade Group for each patient sample was determined by histological analysis of prostate biopsies after MRI and targeted ± systematic biopsies.

Recently, it was shown that PSA levels can be monitored directly from a patient's urine samples which offers a convenient non-invasive alternative also for screening purposes^14^. Measuring PSA or other biomarkers directly from urine is clearly an important track of research. Lately, we succeeded in measuring urinary tract infection directly from clinical samples using the same analytical platform^7^. It is known that concentrations of urine parameters vary a lot within-individuals and between individuals and it is likely that some urine parameters are age related^17^. Another important matter is whether the urine sample is taken before or after prostate biopsy. After biopsy operation, the prostate may leak more potential biomarkers than in an intact state. These are issues that needs to be considered as the quantitative nature of the screening test is examined (Fig. 6).

**Figure 7:**
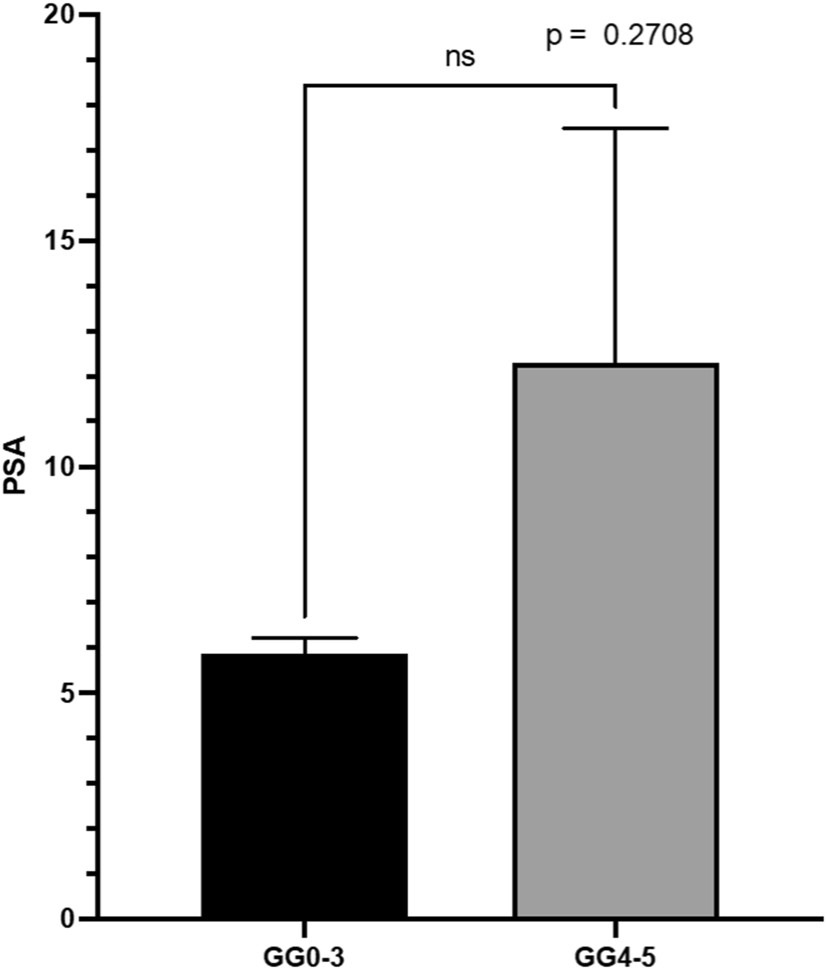
Mean PSA for the GG 0–3 group (left black bar) and the GG 4–5 group (right gray bar). The Y-axis is the linear combination of the measurements (Mean, N = 96, median of 3 replicates, error bars SEM).

All the patients were also tested with PSA-antibody based blood test which is currently the gold standard method. The results were statistically non-significant, which is often seen with lethal vs non-lethal comparisons (Fig. 7). Instead, with our phage-based method the results were clear and the groups differed significantly (Fig. 6).

In this study, the serum PSA levels of the patients with high risk grade groups 4 and 5 varied between 3.1 and 36.8 ng/mL. In patients with grade group 0–3 the serum PSA levels varied between 3.1 and 17.2 ng/mL. Generally, serum PSA-levels less than 4.0 ng/mL are considered normal and clinicians do not prescribe further investigations^18^.

Our search for potential biomarkers is partly based on microplates having high protein and peptide-binding capacities. The highly charged polystyrene well has a high affinity towards polar and hydrophilic molecules that the clinical urine samples have [ThermoFisher Scientific, Waltham, MA, USA]. The biomarker target for our M13 phage is therefore likely a protein or peptide that is passed through to the urinary tract.

## Discussion

We present a novel concept for a screening bioassay. We found that our assay for CRP as a simulant biomarker supported our initial hypothesis of how the assay performs under controlled conditions. The concentration of CRP correlated with the measured reporter luminescence signal and absorbance based on our original assumptions. The more challenging part was to apply the method into classifying lethal PCa against non-lethal ones from patient urine samples. Our assay demonstrates a statistically significant difference between the two groups (p < 0.0014).

The development procedure of the biosensor is straightforward. Biopanned phages with double affinity interact with our quencher dye molecules and biomarkers, changing the quenching capacity of the dye. This can be measured with the modulation of reporter luminescence and in absorbance. The mechanism behind the reaction is probably a result of the changed microenvironment of the dye molecules, affinity towards biomarkers and the resulting order of the phage network.

Our novel screening method demonstration utilized biomarker samples that were easily and noninvasively obtained, making sampling quick and pleasant for the patient and potentially reducing the need for special expertise and workload if applied to mass screening. To validate this, we aim to screen a much higher number of patient samples in the next step of our project. Furthermore, urine as a sample matrix requires a special consideration due to its varying concentration of molecules. The possibility of non-invasive screening and diagnostic methods have garnered attention lately due to their undeniable benefits. Additionally, our method has a potential in finding novel disease biomarkers. Enriching specific phage-bound biomarkers will narrow down the variety of candidate molecules and the biomarker selection can be controlled.

Although the number of potentially lethal high-grade PCa samples at our disposal was rather small, the results of our study provide proof-of-concept evidence for the assay for the detection of lethal variants of PCa using urine samples. Differential separation of lethal prostate cancers indicates that our system interacts with molecules unrelated to PSA. PSA is used as a gold standard in prostate cancer diagnostics and our novel biosensing system could be e.g. used alongside PSA testing as a complementary method.
